# Proof of principle for the clinical use of a CE-certified automatic imaging analysis tool in rare diseases studying hereditary spastic paraplegia type 4 (*SPG4)*

**DOI:** 10.1038/s41598-022-25545-z

**Published:** 2022-12-21

**Authors:** Tobias Lindig, Benjamin Bender, Eva Bürkle, Vinod Kumar, Ulrike Ernemann, Ludger Schöls, Tim W. Rattay

**Affiliations:** 1 Department of Diagnostic and Interventional Neuroradiology, University Hospital Tübingen, Hoppe-Seyler-Straße 3, 72076 Tübingen, Germany; 2 AIRAmed GmbH, Konrad‑Adenauer‑Str. 13, 72072 Tübingen, Germany; 3 Department of Neurodegenerative Disease, Hertie-Institute for Clinical Brain Research, and Center for Neurology, University of Tübingen, Tübingen, Germany; 4 German Center of Neurodegenerative Diseases (DZNE), Tübingen, Germany; 5 Center of Rare Diseases (ZSE), University of Tübingen, Tübingen, Germany; 6 Max Planck Institute for Biological Cybernetics, High-Field MR Center, Tübingen, Germany

**Keywords:** Motor neuron disease, Movement disorders, Neurodegeneration, Neurodegenerative diseases

## Abstract

Usage of MR imaging biomarkers is limited to experts. Automatic quantitative reports provide access for clinicians to data analysis. Automated data analysis was tested for usability in a small cohort of patients with hereditary spastic paraplegia type 4 (SPG4). We analyzed 3T MRI 3D-T1 datasets of n = 25 SPG4 patients and matched healthy controls using a commercial segmentation tool (AIRAscore structure 2.0.1) and standard VBM. In SPG4 total brain volume was reduced by 27.6 percentiles (p = 0.001) caused mainly by white matter loss (− 30.8th, p < 0.001) and stable total gray matter compared to controls. Brain volume loss occurred in: midbrain (− 41.5th, p = 0.001), pons (− 36.5th, p = 0.02), hippocampus (− 20.9th, p = 0.002), and gray matter of the cingulate gyrus (− 17.0th, p = 0.02). Ventricular volumes increased as indirect measures of atrophy. Group comparisons using percentiles aligned with results from VBM analyses. Quantitative imaging reports proved to work as an easily accessible, fully automatic screening tool for clinicians, even in a small cohort of a rare genetic disorder. We could delineate the involvement of white matter and specify involved brain regions. Group comparisons using percentiles provide comparable results to VBM analysis and are, therefore, a suitable and simple screening tool for all clinicians with and without in-depth knowledge of image processing.

## Introduction

Hereditary spastic paraplegias (HSP) are neurodegenerative disorders of the first motor neuron with the clinical hallmark of progressive spasticity and weakness of lower limbs^1,2^. Despite being rare diseases with a prevalence of 2–10:100,000^3^, they are highly genetically and phenotypically heterogeneous. Up to date, about 80 genes and loci have been proven to cause HSP^4–9^ with dominant mutations in the *SPAST* gene causing HSP type 4 (SPG4)^10^, which accounts for more than a fourth of all and almost half of genetically defined HSP cases^11^.

Systematic MRI studies in representative HSP cohorts are limited^12^, with SPG4 being the best-studied genotype^13–18^. Routine radiological imaging in HSP is clinically relevant for exclusion of relevant differential diagnosis (e.g. tumors or multiple sclerosis) and considered to be regular despite e.g. some HSP forms where a thinning of the corpus callosum has been found (SPG11^19^ & SPG15^20^ > > SPG35^21^ and other ultra-rare genotypes). In the second most common HSP form with cerebellar ataxia, SPG7, cerebellar atrophy is a frequent finding. In cases where routine imaging findings by visual inspection are considered unremarkable, quantifiable brain atrophy patterns might further guide clinicians to diagnosis within the broad spectrum of neurodegenerative diseases and beyond HSP. Also, quantitative brain measures might prove beneficial for disease monitoring if regional brain volume loss can be detected compared to healthy controls. In recent years, some CE- and FDA-certified software solutions have become available, but evidence of diagnostic performance in patients is currently scarce. Available open-source solutions like SPM12^22^, FreeSurfer^23^, or SIENA^24,25^ may not be used for clinical decision-making. Therefore, we reevaluated our previously published quantitative MRI study in SPG4^14^ with a commercially available CE-certified product to evaluate the clinical use and performance in a known cohort.

## Material and methods

MRI was performed as previously published^14^ on a 3 T whole-body MR system (Skyra, Siemens, Erlangen, Germany) at the University Hospital Tübingen using a 32-channel head coil. Acquired 3D-T1 datasets of adult SPG4 patients (n = 25, clinical details and mutations see^14^ for n = 15 and n = 10 novel cases with confirmed *SPAST* mutations) and healthy age- and sex-matched controls (n = 25) were analyzed using AIRAmed neural network analysis.

### Ethics approval and consent to participate

The study protocol was approved by the medical ethics committee at the University of Tübingen, Germany (reference numbers 833/2016B02 and 690/2011BO1). Informed written consent was obtained from all subjects prior to examinations. The study has been performed by the ethical standards laid down in the 1964 Declaration of Helsinki and its later amendments.

### Automated MRI evaluation

**Figure 1:**
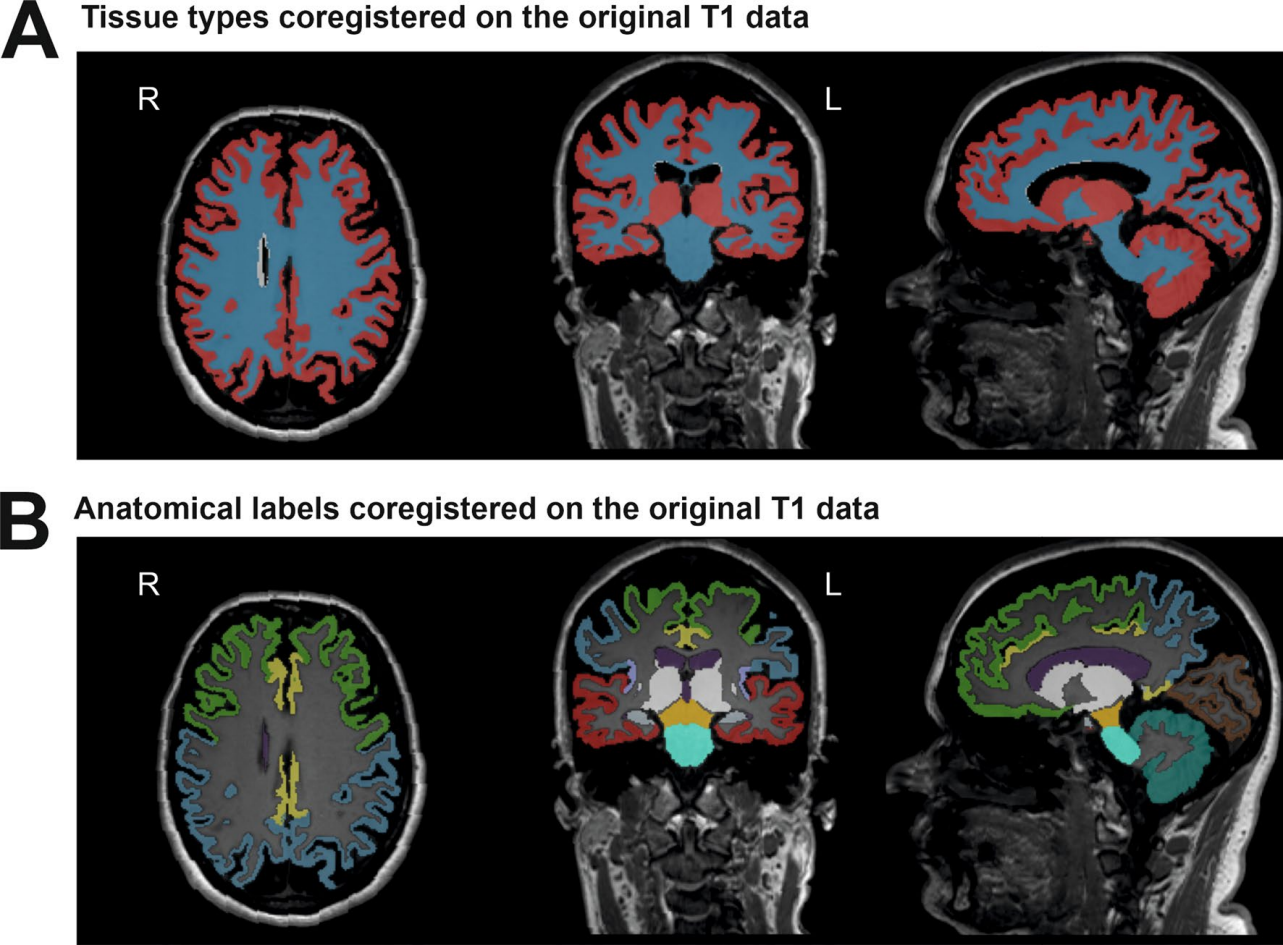
Exemplary images from a report of a SPG4 patient to control the automated segmentation results. These representative images were taken from an AIRAscore structure report of a SPG4 patient (#11) which can be used for quality control of the segmentation process. In (**A**) the segmentation results show the gray matter (in red) and the white matter (in blue) in transversal, coronal, and sagittal planes. In (**B**) the anatomical labels of the specified brain lobes are shown: frontal (green), parietal (blue), temporal (red), occipital (brown), and insula (lilac). Additional regions are also labeled the midbrain (orange), pons (turquoise), cingulate gyrus (yellow), hippocampus (grey), the ventricles (purple), and the cerebellum (darker turquoise).

Before automated MRI evaluation, all images were checked to rule out severe artifacts or incidental findings. Afterward, DICOM data was transferred to an automated segmentation tool AIRAscore structure v2.0.1 (AIRAmed GmbH, Tübingen, Germany) directly from the scanner, which creates a PDF report (Supplementary File 1) and two 3D overlays to control for correct segmentation of anatomy and tissue segmentation by the underlying neuronal networks (3D CNNs), as reported previously^26^. Results are stored in the local PACS and are presented in ml, in % of total intracranial volume, and as percentiles compared to an age- and gender-matched control dataset included within the product that consists of over 8000 healthy controls from a variety of scanners and sequences at 1.5 and 3 T. As percentiles offer the advantage that age, gender, and head size (TIV) based effects are already corrected, the variability on the healthy population is the basis for the underlying distribution they were used for all further evaluations. No other forms of preprocessing or postprocessing are necessary. For research purposes a dedicated interface is available (by request to the manufacturer, not part of the service) that allows an automatic export of the segmentation results in machine readable format. Two neuroradiologists controlled all reports visually (TL, BB) with the accompanying 3D overlay images for correct segmentation results (Fig. 1) before further statistical evaluation. One patient was excluded from the analysis (similar to VBM analysis in^14^) because of incorrect segmentation; subsequently, the matching control was also excluded. This incorrect automated segmentation was visible on the reports (Fig. 1 and Supplementary File 1) and easily recognizable for clinicians.

For comparison and external evidence, the same data was also evaluated with a classical voxel-based morphometry analysis (VBM) using the computational anatomy toolbox CAT12 (http://www.neuro.unijena.de/cat/, v12.8.1) for statistical parametric mapping software SPM12 (v7771) of the Wellcome Department of Cognitive Neurology (Supplementary File 2).

### Statistical analysis

Statistics were performed using SPSS 28 (IBM, Armonk, NY). Gaussian distribution of parameters was assessed using the Shapiro–Wilk-Test. Gaussian distributed data are presented as mean (standard deviation) and non-gaussian distributed data as median^$^ {interquartile range}. Gaussian distributed variables were compared by a two-sided t-test, non-Gaussian distributed variables by the Mann–Whitney-U test, and nominal variables by the Chi-square test.

Benjamini & Hochberg (B&H) procedure^27,28^ has been applied to correct for multiple testing and to control the false discovery rate when analyzing all primary outcome parameters. Only corrected p-values are reported. The analysis was a two-step process; initially, the percentiles of three parameters (total brain volume, gray matter, and white matter) and then the percentiles of the different brain areas or indirect markers, the percentiles of ventricular sizes, were compared (total N = 24). All other comparisons represent exploratory analyses; no correction for multiple testing was applied.

The effect sizes (r) were calculated according to $r=\left|\frac{Z}{\sqrt{n}}\right|$ with r > 0.1 < 0.3 considered as small, r > 0.3 < 0.5 as medium and r > 0.5 as large.

As an exploratory approach, correlations of the primary outcome parameters with age, disease duration, and SPRS total score were analyzed with the Pearson or Spearman-Rho correlation coefficients (depending on Gaussian data distribution) not corrected for multiple comparisons.

## Results

### Demographics

There were no significant group differences (Table 1) for age with thirteen male and eleven female SPG4 patients and age- and gender-matched healthy controls. The median disease duration of the SPG4 cohort (n = 24) was 15.0 years [range: 2–70 years] with a mean age of 50.4 years. The disease severity was assessed by the Spastic Paraplegia Rating Scale (SPRS)^29^, with a median of 20.5 points.

**Table 1:** Demographics of the analyzed SPG4 cohort and sex- and age-matched controls.

		Healthy controls (n = 24)	SPG4 patients (n = 24)	p-values
Gender	♀	11	11	**1.0**
	♂	13	13	
Age [years]		49.8 (11.59)	50.4 (11.52)	0.852
Disease duration [years]^$^		n/a	15.0^$^ {19.0}	
SPRS total score^$^		n/a	20.5^$^ {21}	

Presented are demographics as well as standardized disease parameters of the SPG4 patients and their age- and gender matched controls. Gaussian distribution of parameters was assessed using the Shapiro–Wilk-Test. Age as Gaussian distributed variable was compared by a two-sided t-test, disease duration and total SPRS score (non-Gaussian distributed variable) were tested using the Mann–Whitney-U test, and gender as nominal variable with chi square test. Gaussian distributed data is presented as mean (standard deviation) and non-gaussian distributed data (as median^**$**^ {interquartile range}.

Significant values are in bold.

^$^Non-gaussian distributed variable, n/a not applicable**.**

### Volume percentiles

**Figure 2:**
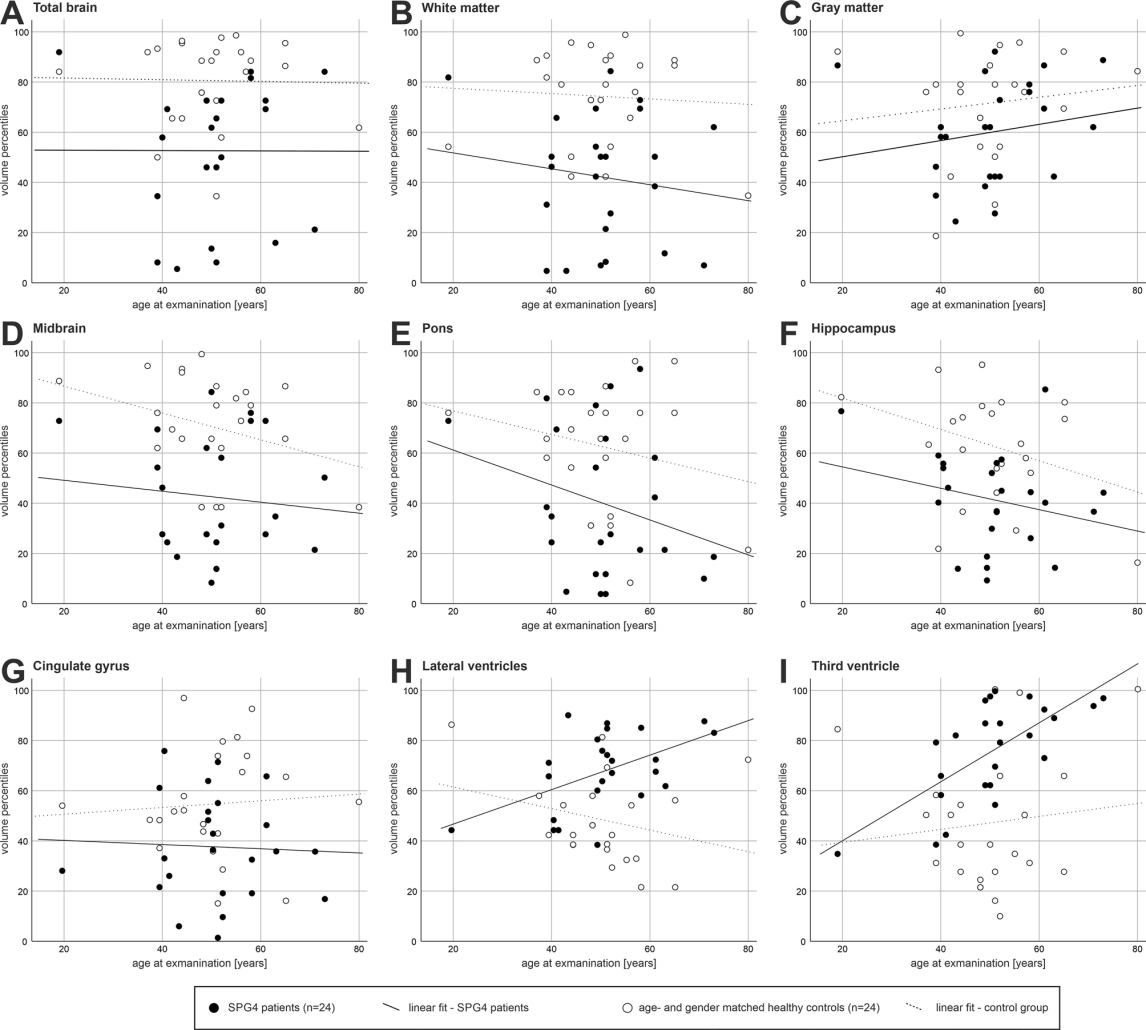
Scoring diagram of all percentile values of the SPG4 cohort and their age- and gender-matched controls are shown with their age dependency. All percentile values (y-axis) of the SPG4 patients (filled black dots) and the controls (unfilled dots) are shown together with the respective age of the individual (x-axis) for representative brain region: (**A**) Total brain, (**B**) White matter, (**C**) Gray matter, (**D**) Midbrain, (**E**) Pons, (**F**) Hippocampus bilateral, (**G**) Cingulate gyrus bilateral, (**H**) Lateral ventricles, and (**I**) Third ventricle. In all of these shown areas but Gray matter in (**C**) were significant group differences (compare Table 2). Linear fits are shown for the controls (dotted line) and the SPG4 patients (black line).

First-level analysis (Table 2) revealed a significant reduction in total brain volume of 27.6 percentiles (p = 0.001), caused mainly by a reduction in white matter (30.8 percentiles, p < 0.001) in SPG4 patients compared to controls. There was no significant reduction in total gray matter. In a second step, specified brain regions were analyzed (Table 2) or specific structures like the ventricular system. There was no significant reduction in the cerebellar volume or the cerebellar gray matter and not in the cerebral gray matter in SPG4 patients. A significant reduction of the midbrain volume (41.5 percentiles, p = 0.001) was found as well as a significant reduction of pons volume (36.5 percentiles, p = 0.020) with a stable midbrain/pons ratio indicating a similar reduction of both brainstem regions. There were no changes in the cortical gray matter of brain lobes (frontal, parietal, temporal, or occipital lobe) or the insula region (for details, see Table 2). The hippocampal volume was reduced by 20.9 percentiles in SPG4 patients (p = 0.002) with a gray matter reduction of the cingulate gyrus (− 17.0 percentiles; p = 0.020). Both lateral ventricles (19.3 percentiles; p = 0.001) and the third ventricle (40 percentiles; p = 0.002) were significantly enlarged in the ventricular system, while the fourth ventricle was unchanged. The effect sizes were large (see Table 2). Scoring diagrams of all percentile values are presented in Fig. 2, including linear fits for parameters with significant group differences.

**Table 2:** Group-level results of AIRAscore-reported brain area volume percentiles of SPG4 patients and matched healthy controls.

Specified area	Percentiles Mean (SD) or median^$^ {IQR}		p-values (B&H)	Effect size $$r=\left|\frac{Z}{\sqrt{n}}\right|$$
	Healthy controls (n = 24)	SPG4 patients (n = 24)		
**Total brain**^**$**^	87.45^$^ {25.7}	59.85^$^ {48.1}	**0.001**	**0.788**
cerebellum^$^	81.6^$^ {31.5}	80.2^$^ {37.0}	0.794	0.075
**Gray matter (GM)**	71.31 (20.70)	59.81 (20.45)	0.112	0.404
GM cerebrum	67.97 (20.80)	59.20 (19.45)	0.225	0.349
GM cerebellum^$^	75.3^$^ {32.04}	83.78^$^ {30.48}	0.848	0.027
White matter (WM)^$^	78.80^$^ {31.6}	48.0^$^ {50.70}	** < 0.001**	**0.845**
**Infratentorial**				
Midbrain^$^	74.2^$^ {24.6}	32.7^$^ {43.1}	**0.001**	**0.792**
Pons^$^	67.4^$^ {42.6}	30.95^$^ {55.1}	**0.020**	**0.537**
Midbrain/pons ratio^$^	57.9^$^ {29.2}	65.50^$^ {63.8}	0.446	0.189
**Lobe**				
Frontal	63.88 (23.46)	54.71 (23.92)	0.272	0.305
Parietal	55.18 (23.33)	47.64 (27.30)	0.419	0.250
Temporal	59.17 (25.18)	49.13 (21.25)	0.225	0.334
Occipital	46.48 (25.64)	47.06 (25.59)	0.848	0.002
Insula	38.43 (25.17)	39.49 (21.25)	0.848	0.050
**Limbic system**				
Hippocampus (bilat.)	63.0 (22.01)	42.13^$^ {28.40}	**0.002**	**0.667**
GM cingulate gyrus	54.44 (21.33)	37.41 (20.96)	**0.020**	**0.532**
**Ventricular system**				
Lateral ventricles	48.31 (17.44)	67.58 (15.47)	**0.001**	**0.734**
Third ventricle^$^	38.2^$^ {36.2}	80.2^$^ {31.2}	**0.002**	**0.705**
Fourth ventricle	40.15 (21.95)	53.2 (20.83)	0.085	0.442

Presented are percentiles of brain volumes as reported by AIRAscore and age- and gender matched healthy controls (n = 24 each). Gaussian distribution of parameters was assessed using the Shapiro–Wilk-Test. Gaussian distributed data (gray matter, GM cerebrum, lateral ventricles, fourth ventricle, all lobes, hippocampus, and GM cingulate gyrus) is presented as mean (standard deviation) and non-gaussian distributed data^**$**^ as median^**$**^ {interquartile range}. Gaussian distributed variables were compared by a two-sided t-test and non-Gaussian distributed variables by the Mann–Whitney-U test. Reported p-values are corrected after Benjamini&Hochberg (B&H) with p-values < 0.05 reported in bold. Effect size values r > 0.1 < 0.3 were considered as small, r > 0.3 < 0.5 as medium and r > 0.5 as large, all large effect sizes are reported in bold.

^$^ non-gaussian distributed variable, bilat.: bilateral; GM: gray matter; IQR: interquartile range; SD: standard deviation.

### VBM analysis and SPM12 correlation

Voxel-based analysis of the gray matter (Supplementary File 2A) showed symmetric volume reduction in the mediodorsal thalamus and in paravermal and vermal areas of lobule 5, 6, and 7 of the cerebellum for uncorrected p < 0.001; for FEW corrected p < 0.05 only in the pulvinar of the right thalamus a significant volume reduction remained. Voxel-based analysis of the white matter (Supplementary File 2B) revealed extended symmetric volume reduction in the deep white matter, the corpus callosum, and to a lesser extent in the brainstem for uncorrected p < 0.001; for FEW corrected p < 0.05 only in the periventricular white matter and the corpus callosum a significant volume reduction remained.

Absolute and relative (in %TIV) WM, GM, brain volumes, and the total TIV estimates of SPM12 segmentation and AIRAscore were correlated. The absolute volumes had a very strong correlation for all measures (GM r = 0.948, WM r = 0.985, brain volume r = 0.978, TIV r = 0.983), while relative volumes had lower but still strong correlations (GM r = 0.746, WM r = 0.915, brain volume r = 0.808).

Independent t-test between controls and patients for relative GM, WM and brain volume yielded a significant difference for WM (p < 0.001) and brain volume (p = 0.015) with SPM12 and for WM (p < 0.001) and brain volume (p = 0.002) with AIRAscore based on %TIV, that was higher with percentiles (WM p < 0.001, brain volume p = 0.001).

### Correlation of imaging results with clinical data

When analyzing the SPG4 cohort in an exploratory approach (not corrected for multiple testing), there was a significant correlation for age with lateral ventricle size (r_P_ = 0.506, p = 0.012) and third ventricle (r_S_ = 0.599, p = 0.002) both not seen in the control cohort (lateral ventricles (r_P_ = − 0.367, p = 0.077); third ventricle (r_S_ = 0.063, p = 0.771)). No correlation with age was found for any volume parameter within the control cohort. There was a significant correlation of the percentiles of the third ventricle with disease duration (r_P_ = 0.448; p = 0.028) but no correlations were found for the disease severity as measured by the SPRS.

## Discussion

Routine radiological imaging by visual inspection in hereditary spastic paraplegia is expected to be unaffected and is mainly performed to exclude relevant differential diagnoses like inflammation or tumors causing spasticity. In our previous study, we already showed a widespread affection of gray and white matter in SPG4. These changes were extensive and unexpected given the relatively pure HSP phenotype, according to Harding^2^. The present study aimed to prove the principle of CE-certified automatic imaging analysis as software as a service (SaaS). We used the AIRAscore evaluation as an easily accessible approach for clinicians to firstly confirm the widespread white matter involvement as previously shown in our DTI study^14^ and others^12,13,17^ and to further delineate involved brain areas in hereditary spastic paraplegia with underlying *SPAST* mutations. Quality control was easily done using the segmentation images (compare Fig. 1), and one case (4%) was mis-segmented, which could be easily detected. In line with a reduction of total white matter, AIRAscore reveals an increase in the volume of the lateral ventricles. Volume reduction of the third ventricle could be a sign of thalamic atrophy. White matter changes (analyzed with DTI) have also been found in other motor neuron diseases like amyotrophic lateral sclerosis^30^. The reduction of total gray matter was unremarkable, in line with our previous results of VBM^14^, which showed less extensive structural changes than DTI in SPG4. On the contrary, regional cerebellar atrophy, as demonstrated by VBM analyses, was not detected by the AIRAscore since no subcerebellar structures are included. A novel finding compared to our previous study and the current VBM analysis (Supplementary File 2) was the reduction of hippocampal volume in SPG4, confirmed in one other recent study^12^. This is an exciting finding concerning the ongoing discussion about cognitive involvement in SPG4, with variable findings depending on study cohorts and test batteries ranging from normal cognitive performance to dementia^31–36^. Interestingly, the cingulum is affected in several imaging studies in manifest SPG4^12,13,36,37^ and was also detected in the automatic AIRAscore analysis, although the SPG4 patients in our previous cohort did not present with apparent cognitive involvement^14^. Cingulum changes occur early in the disease as we were recently able to show changes in the cingulum in prodromal SPG4^26^. The preSPG4 study included a tablet-based neurocognitive assessment using CANTAB™, which did not reveal any abnormalities—similar to the findings in manifest SPG4 in a subgroup of the non-motor symptoms in SPG4 study^36^. However, detailed neuropsychological testing in the entire cohort of all (n = 24) manifest SPG4 patients of this study was not done, and therefore minor neuropsychological deficits might be missed. We also showed a reduction of the midbrain volume and the pons. This might well derive from degeneration of the pyramidal tract in the cerebral peduncles, as demonstrated by our previously published DTI findings^36^.

The lack of correlations of volume percentiles with age in the control group is expected. The reported percentiles in the AIRAscore report are from an age and gender-matched reference cohort, any correlation with age in a healthy population, would suggest an error in the reference set of AIRAscore. Therefore, the correlations with age are robust if they are found in the disease group (lateral ventricles and third ventricle) as indirect correlates of brain atrophy due to disease duration. Accordingly, we could show a correlation between disease duration and the enlarged third ventricle volume that is in line with the atrophy of the thalamus^12,14,38^. Unfortunately, deep gray matter structures, including the thalamus, are not available in AIRAscore until the next release. It would be exciting to look at the correlation for disease duration and severity here.

In general, on the individual level, sensitive percentile values can not discriminate patients from healthy controls (as seen in Fig. 2) in SPG4 since these structural differences are very small with overlap between both groups. However, some parameters are better discriminators than others (e.g. hippocampus and lateral ventricles). In order to establish cut-off or predictive values, for example, which could be used in the diagnostic process to distinguish different HSP genotypes to steer genetic testing, comparative analyses between HSP genotypes are needed.

Whether AIRAscore detected changes are suitable as a quantitative measure for the degenerative process will require prospective longitudinal MRI studies. Early and presymptomatic patients need to be studied to see if, when and at which rate changes occur. One limitation of the current study is the missing correlation with disease severity scores like the SPRS. This might be because most patients were already in a moderately advanced disease stage (median SPRS score 20.5 and disease duration of 15.0 years), and only a limited number of patients with an SPRS < 7 (n = 5) or a disease duration < 10 years (n = 5) were included in this study. Cognitive performance results were unavailable in the cohort, therefore, a specific correlation for the hippocampal volume was not possible. More detailed clinical scores are needed that test specific subfunctions that have an anatomic link with the current findings, e.g. cognitive function and hippocampal volume to provide more specific clinical correlates than disease duration in the future.

While a large reference dataset within a software offers some advantages, it also has some limitations. Namely, the underlying distribution will be broadened due to scanner and sequence effects, which can lead to false negative individual cases and a small systematic shift of the percentiles. Therefore, follow-up examinations still need to be measured with the same protocol and same scanner. Still, the fast processing time of less than 5 min, the easy availability without any need for preprocessing, and the ability to check segmentation results within the PACS offer several advantages over standard scientific applications that are also not certified for clinical use.

AIRAscore provides us with a tool that enables the detection of even small changes in brain volume earlier and in more detail than by visual assessment or state-of-the-art VBM analysis alone. The comparison with a large reference population allows us to objectively distinguish between pathological and healthy. Group comparisons using percentile values align with results from VBM analyses and are a simple screening tool for research without in-depth knowledge of image processing.

## Conclusion

Quantitative imaging reports proved an easily accessible screening tool for small cohorts to delineate the involvement of gray or white matter and specific brain regions. Since the reports provided do not require detailed expertise for image analysis, certified automatic volumetry tools seem to be exceedingly feasible also for clinicians. Group comparisons using percentiles allow us to confirm or predict results from VBM analyses. Even on an individual level, thanks to the extensive reference group of healthy adults, the use of percentiles as guidance for any neurodegenerative conditions could be beneficial, not limited to HSP.

## Supplementary Information

Supplementary Information 1.

Supplementary Information 2.

## Data Availability

The data sets for this manuscript are not publicly available because raw data regarding human subjects (e.g., genetic raw data, personal data) are not shared freely to protect the privacy of the human subjects involved in this study; no consent for open sharing has been obtained. Requests to access an anonymous imaging data set should be directed to Tobias Lindig.
