# Supplementary material for: Proof of principle for the clinical use of a CE-certified automatic imaging analysis tool in rare diseases studying hereditary spastic paraplegia type 4 (SPG4)
Source: Sci Rep. 2022 Dec 21;12:22075. doi: 10.1038/s41598-022-25545-z (PMC9772173; doi:10.1038/s41598-022-25545-z)
Supplement: Supplementary file 1 — Supplementary Information 1. [file 41598_2022_25545_MOESM1_ESM.pdf]

AIRAscore structure

Report

AIRAmed
artificial intelligence in radiology

Order-ID:	468 JLU 28K
Person:	**SPG4 example** (f), born Mar 01, 1971
Acquisition:	**Feb 28, 2015**, 10:38 AM
Evaluation:	May 04, 2022, 07:53 AM (+02:00 UTC), Software Version 2.0.1
Sequence:	3, t1_mprage_sag_p2_iso_0.9, sagittal, 0.9 × 0.9 × 0.9 mm³, TR: 2300.0 ms, TE: 2.32 ms, TI: 900.0 ms, FA: 8.0°, SIEMENS, Skyra, 3.0 T

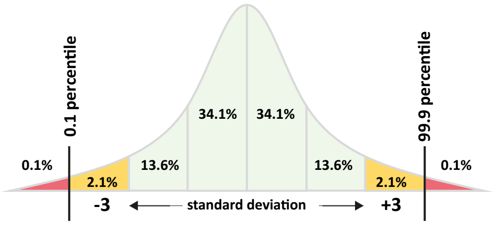

	absolute volume in ml	relative volume in %TIV	(percentile)	(normal range[1] in ml)
**Tissue Type**				
**Total brain volume**	1054.1	73.5	(5.5)	(1036.1 - 1215.9)
**Gray matter** (GM)	614.1	42.8	(24.2)	([illegible]74.9 - 697.9)
**White matter** (WM)	437.9	30.5	(4.5)	(430.1 - 550.6)
GM **cerebrum (L)**	229.5	16.0	(18.4)	(214.8 - 267.1)
GM **cerebrum (R)**	230.4	16.1	(21.2)	(214.6 - 267.6)
**T1 hypointense lesions** (white matter lesions, perivascular spaces)	2.17 (pathol. > 1)	0.15		
**Infratentorial**				
**Midbrain**	5.5	0.4	(18.4)	(5.0 - 6.7)
**Pons**	11.9	0.8	(4.5)	(11.5 - 17.7)
**Midbrain / Pons**	0.46#		(96.4)	(0.3358 - 0.4666)
**Total cerebellum**	135.3	9.4	(54.0)	(112.2 - 156.8)
GM **cerebellum (L)**	55.7	3.9	(57.9)	(45.0 - 64.0)
GM **cerebellum (R)**	55.7	3.9	(57.9)	(45.0 - 64.0)

[1] Normal range is adapted for head size, age and gender
Without unit

⚠ **The evaluation results of this report must be checked for plausibility and correctness by a qualified physician (specialist) for clinical assessment and diagnosis.**

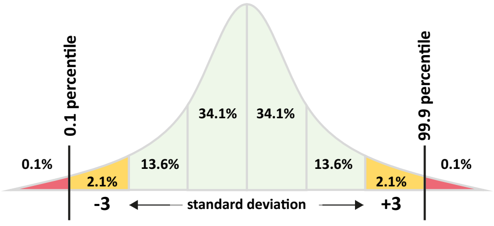

	absolute volume in ml	relative volume in %TIV	(percentile)	(normal range[1] in ml)
**Ventricles**				
**Lateral ventricle (L)**	15.1	1.1	(86.4)	(0.0 - 22.1)
**Lateral ventricle (R)**	16.5	1.2	(93.3)	(0.0 - 20.0)
**3rd ventricle**	1.22	0.08	(81.6)	(0.0 - 1.8)
**4th ventricle**	1.5	0.1	(42.1)	(0.[illegible] - 2.7)
**Brain Lobes** (gray matter)				
**Frontal (L)**	81.3	5.7	(27.4)	(73.9 - 95.2)
**Parietal (L)**	55.7	3.9	(61.8)	(46.9 - 6[illegible].0)
**Occipital (L)**	22.3	1.6	(13.6)	(20.2 - 29.9)
**Temporal (L)**	51.8	3.6	(15.9)	(48.0 - 63.7)
**Insula (L)**	6.3	0.4	(2.9)	(6.2 - 8.7)
**Frontal (R)**	79.0	5.5	(18.4)	(73.3 - 94.7)
**Parietal (R)**	57.4	4.0	(72.6)	(47.5 - 62.8)
**Occipital (R)**	24.8	1.7	(27.4)	(21.2 - 31.4)
**Temporal (R)**	51.7	3.6	(18.4)	(47.4 - 63.2)
**Insula (R)**	6.6	0.5	(13.6)	(6.0 - 8.5)
**Limbic Lobe**				
**Hippocampus (L)**	3.14	0.22	(15.9)	(2.8 - 4.2)
GM **cingulate gyrus (L)**	9.0	0.6	(9.7)	(8.3 - 12.0)
**Hippocampus (R)**	3.13	0.22	(11.5)	(2.8 - 4.4)
GM **cingulate gyrus (R)**	7.8	0.5	(1.8)	(7.9 - 11.2)

[1] Normal range is adapted for head size, age and gender

⚠ **The evaluation results of this report must be checked for plausibility and correctness by a qualified physician (specialist) for clinical assessment and diagnosis.**

Order-ID: 468 JLU 28K
Person: **SPG4 example** (f), born Mar 01, 1971
Acquisition: **Feb 28, 2015**, 10:38 AM
Evaluation: May 04, 2022, 07:53 AM (+02:00 UTC), Software Version 2.0.1

Brain Volume Reduction:

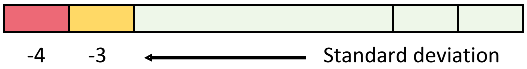

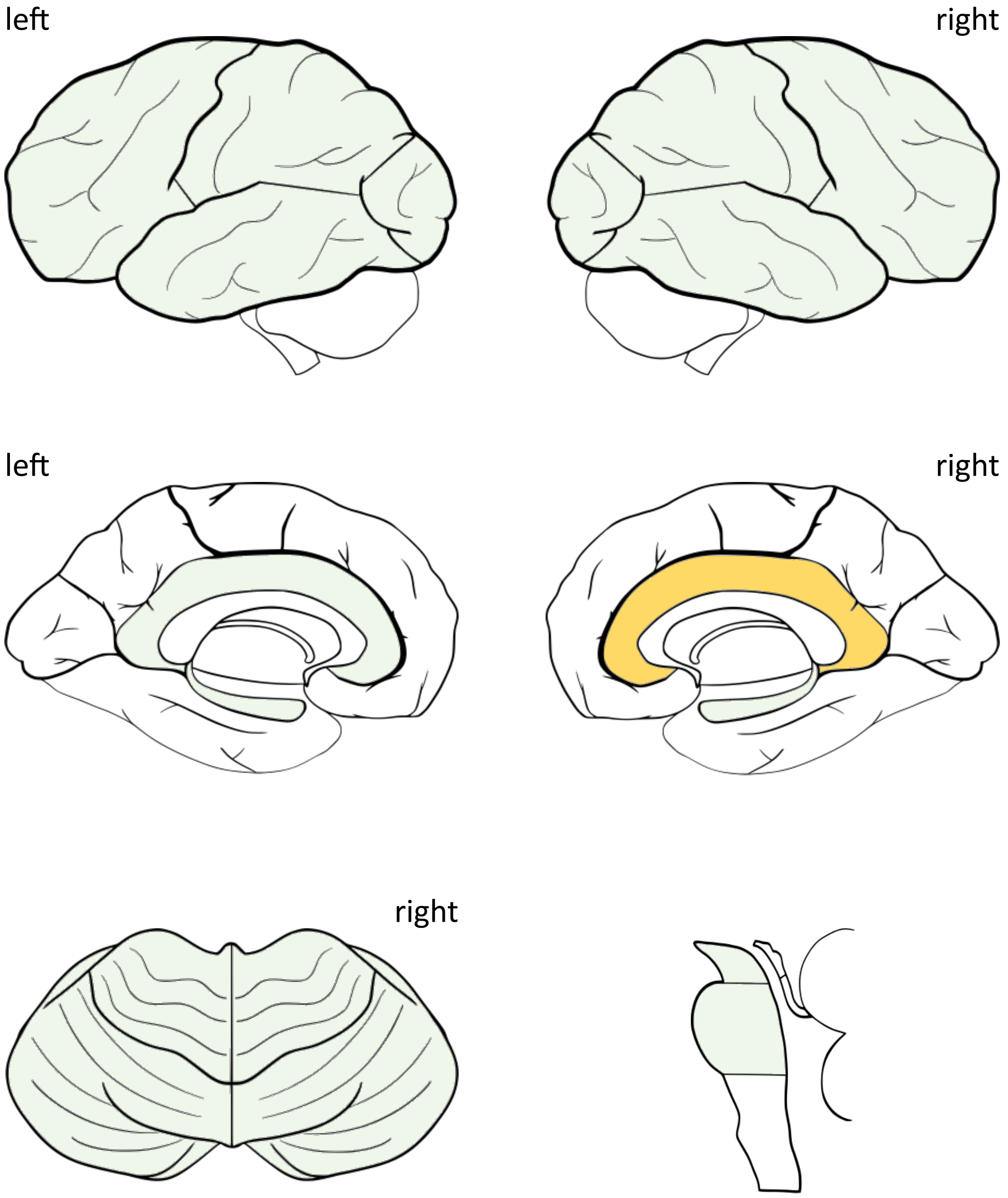

⚠ **The evaluation results of this report must be checked for plausibility and correctness by a qualified physician (specialist) for clinical assessment and diagnosis.**

Standardized Slices for Validation:

Tissue classes coregistered to the original T1 image

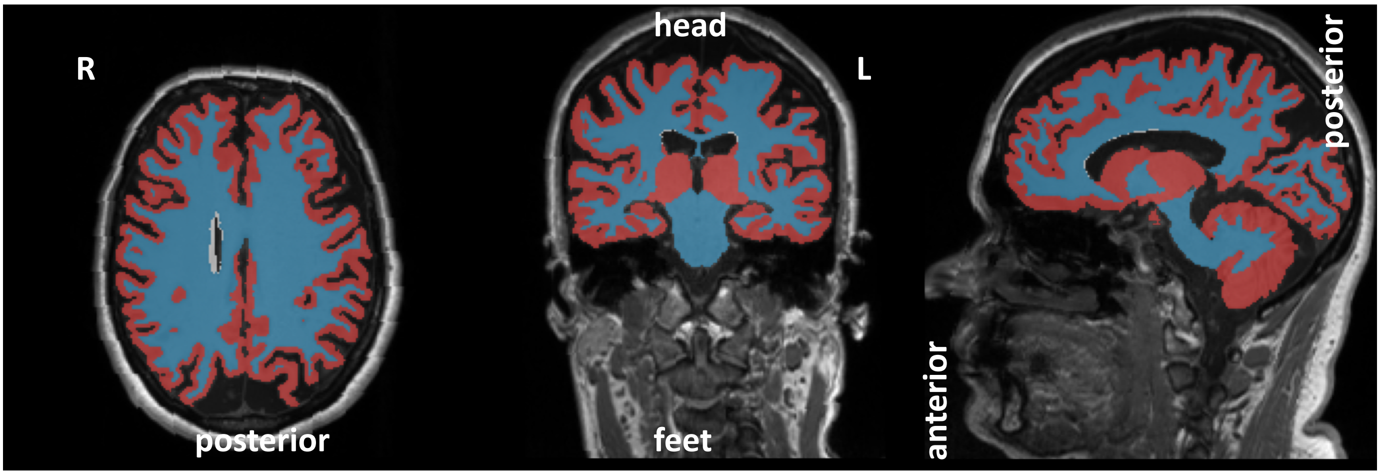

Anatomic labels coregistered to the original T1 image

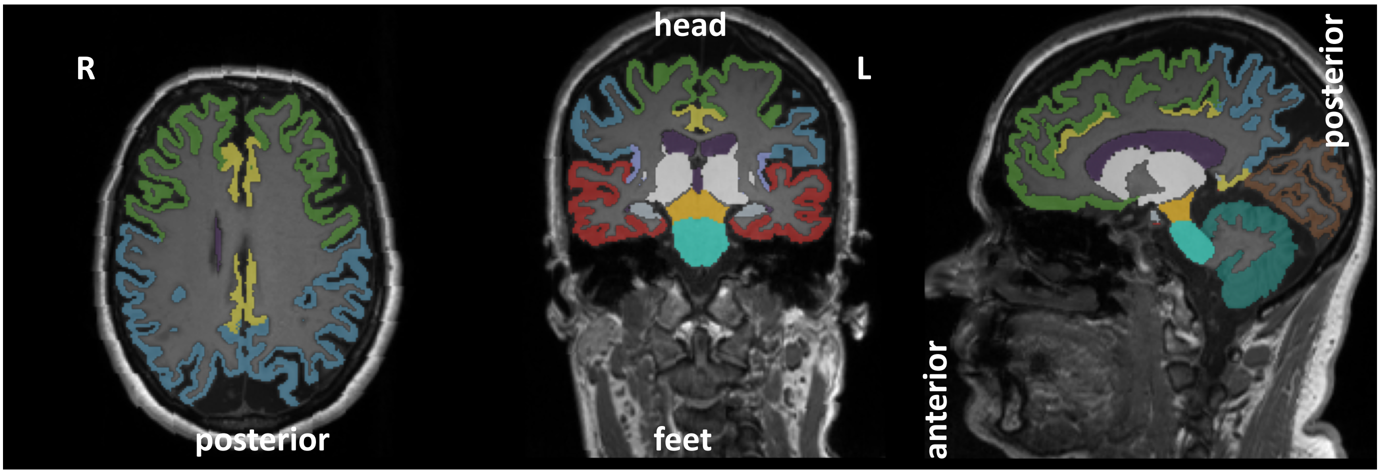

The segmented tissue classes and anatomical labels must be correctly displayed on the anatomy of the original T1 image.

⚠ **The evaluation results of this report must be checked for plausibility and correctness by a qualified physician (specialist) for clinical assessment and diagnosis.**

Customer Notice

Dear Customer,

Thank you for choosing AIRAscore.

We would like to point out that a comparability of the present evaluation results cannot be guaranteed if the underlying image data originate from different MR scanners or were acquired with different MR sequences.

The quantitative evaluation of brain structures can only support, but not replace, the assessment and diagnosis by a qualified physician (specialist).

Regarding the lobe anatomy, we point out that cingulum and hippocampus are reported as separate structures. These are not included in the gray matter volume data for the frontal, parietal and temporal lobes.

The measurement accuracy of the reported volumes given in milliliters (ml) is ±0.01 ml.

AIRAmed does not give any warranty for the assessment and diagnosis derived from the results of the report.

The results of this report must be checked by a qualified physician (specialist) for plausibility and correctness before being used for clinical evaluation and diagnosis. Further instructions regarding this can be found in our instructions for use.

To receive a printed version of the instructions for use within 7 days, please contact service@airamed.de.

Do you have any questions or suggestions? As a young, medium-sized company with a university background, we are always open to and grateful for suggestions for improvement.
