# Supplementary material for: Proof of principle for the clinical use of a CE-certified automatic imaging analysis tool in rare diseases studying hereditary spastic paraplegia type 4 (SPG4)
Source: Sci Rep. 2022 Dec 21;12:22075. doi: 10.1038/s41598-022-25545-z (PMC9772173; doi:10.1038/s41598-022-25545-z)
Supplement: Supplementary file 2 — Supplementary Information 2. [file 41598_2022_25545_MOESM2_ESM.pdf]

Proof of principle for the clinical use of a CE-certified automatic imaging analysis tool in rare diseases studying hereditary spastic paraplegia type 4 (*SPG4)*

Supplementary File 2: VBM analysis

Material and Methods:
The NIFTI formatted structural scans were segmented using the computational anatomy toolbox CAT12 (http://www.neuro.unijena.de/cat/) for SPM (https://www.fil.ion.ucl.ac.uk/spm/software/spm12/). In the next step, a smoothing kernel of 8 mm was applied to the output of CAT12, i.e., gray and white matter normalized images. Furthermore, TIV values for each brain were extracted from the CAT12 report XML file. Finally, a statistical comparison between controls and patients (two-sample t-test; age, gender, and TIV adjusted) was performed separately for gray and white matter. The T contrast of control-patient was examined using a statistical threshold of FWE ($p < 0.05$) and uncorrected ($p < 0.001$) for gray and white matter changes.
Results of significantly decreased gray and white matter volumes in SPG4 patients compared to healthy controls are displayed as colour overlays on top of MNI152NLin2009cAsym T1 template.

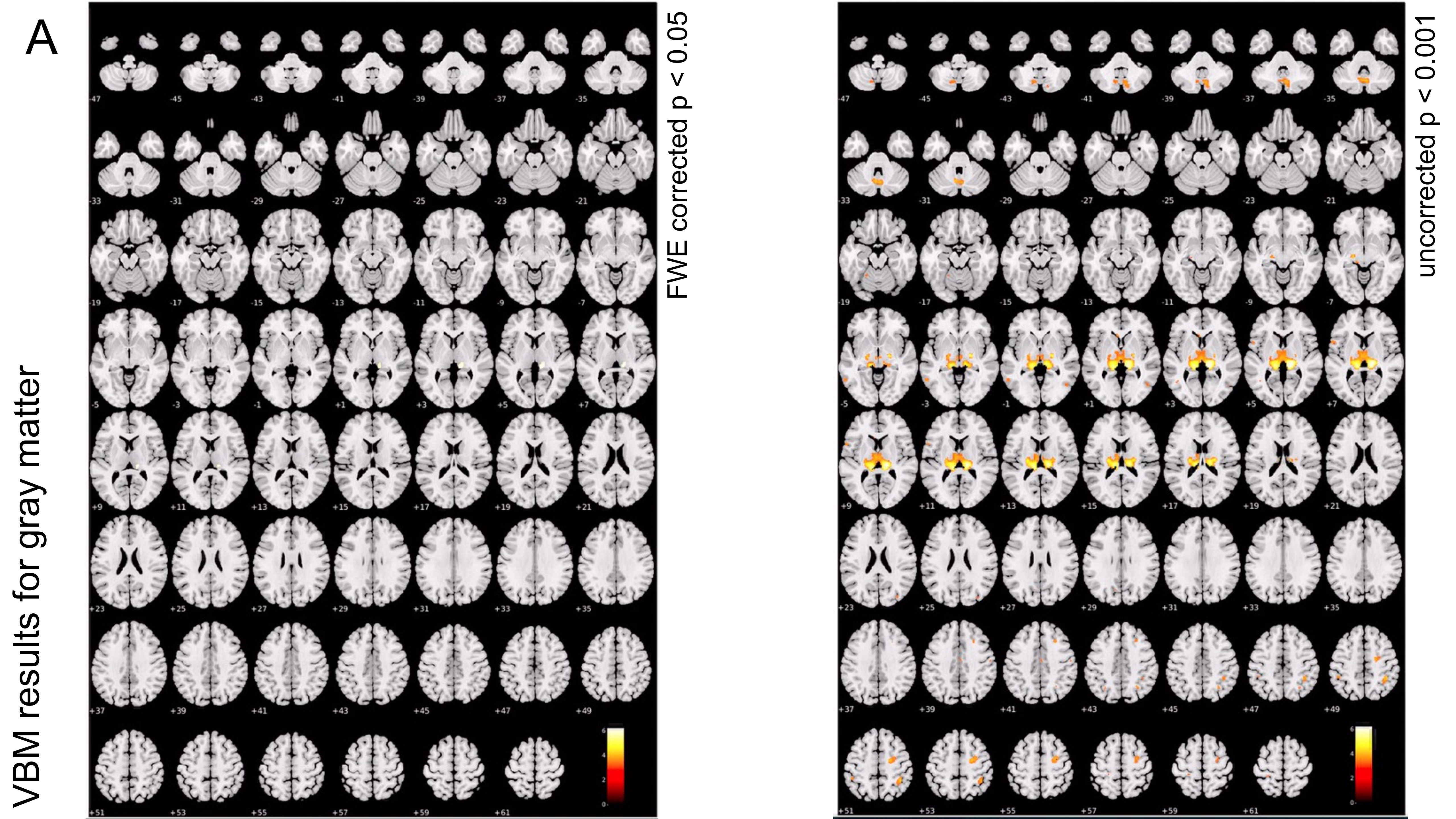
A
VBM results for gray matter
FWE corrected p < 0.05
uncorrected p < 0.001

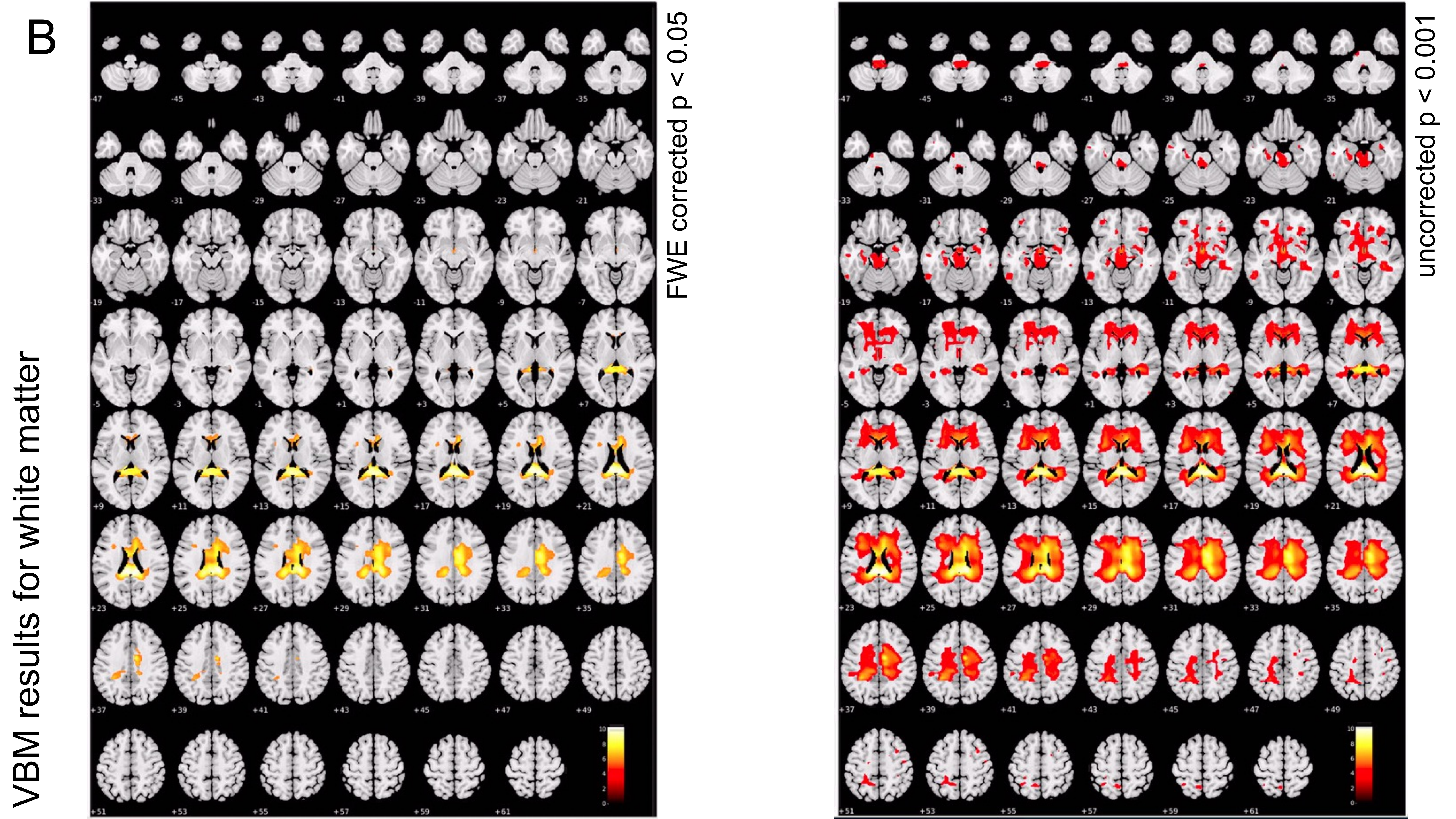
B
VBM results for white matter
FWE corrected p < 0.05
uncorrected p < 0.001
